# Supplementary material for: Does innovation policy drive rural revitalization in China? - An empirical study based on Chinese rural areas
Source: PLoS One. 2025 Apr 7;20(4):e0311462. doi: 10.1371/journal.pone.0311462 (PMC11975105; doi:10.1371/journal.pone.0311462)
Supplement: Attachment — Submitted filename: Review comments.pdf [file pone.0311462.s002.pdf]

Rural revitalization needs the support of innovation policy, and improving innovation capacity is also an important aspect of rural revitalization. The paper has a good value of topic selection, the empirical analysis is more comprehensive, and the overall conclusions are more reliable, but there are still some problems, and this paper suggests a major revision:

1, the introduction part is not refined enough, and it does not highlight the research theme. The introduction uses a lot of language to elaborate the value of innovation, the impact of innovation on the country and society, and the research focus of this thesis is the impact of innovation on rural revitalization, the first half of the introduction does not elaborate the interaction relationship between innovation and rural revitalization, as well as domestic and foreign scholars for the innovation-driven rural revitalization of the theoretical and practical explorations, and the article only has the fifth paragraph briefly explains the impact of innovation on the rural areas, which is obviously insufficient. Meanwhile, the research purpose of this thesis is to argue the impact of national innovative city policy on rural revitalization, in which the transmission of urban innovation capacity to rural areas, that is, the role of urban-rural integration, is particularly important, but it is not mentioned in the introduction. In addition, some expressions in the introduction are inaccurate, for example: “The ongoing process of urbanization is placing significant pressure on rural areas in China, resulting in a range of developmental challenges.” In fact, the impacts of urbanization on rural areas are both beneficial and interdependent. The economic, demographic, and social problems in the Chinese countryside are multifaceted, and the Chinese government's “Rural Revitalization Plan” is not just about solving the problems of population loss and aging in the countryside. This thesis is about the impact of innovation on the revitalization of the rural areas in China, not about why it is necessary to revitalize the countryside, or what problems exist in rural areas in China. It is recommended to carefully sort out the idea of introduction and elaborate the introduction part around the research topic.

2. In “The Dependent Variable $Rural\_indexi$, (Rural Revitalization Index)”, the text does not list in detail the calculation methods of the five subsystem indicators of rural revitalization and their weights. In the section “The Core Explanatory Variable $Inno\_Policyi$, (Innovation Policy)” section, the text similarly does not detail which cities were included in the National Innovation City at what time.

3. For historical reasons, China's cities and villages used to have a long period of time of “urban-rural dichotomy”, and although there have been some improvements in recent years, the government's support for agriculture, rural areas, and farmers, as well as the city's assistance to the countryside, has always been an important aspect of the revitalization of the countryside. For example, the proportion of government financial expenditure on agriculture, forestry and water affairs, the proportion of farmers' transfer income, the level of urban-rural integration, and the structure of the three rural industries, rural industrial structure and other indicators. The text seems to lack the impact of innovation policies on the above indicators, or the impact of the above indicators on rural revitalization, or the role of the above indicators in the promotion of rural revitalization by innovation policies.

4. The year is not specified in the part of data sources.

5. In the part of heterogeneity analysis, the article assigns key cities (provincial capitals and municipalities directly under the central government) a value of 1, and ordinary cities a value of 0. But in fact, in addition to provincial capitals and municipalities directly under the central government, there are also some other national important cities in China, such as sub-provincial

cities, cities under the separate municipalities plan, national central cities, first-tier cities, etc. Some of these cities are not provincial capitals or municipalities, but are also very important in the country, such as Dalian, Xiamen, Qingdao, etc. It is suggested that these cities be included in the “key cities”.

6. The discussion on innovation policies for rural revitalization should be more revealing in the section on “Conclusions and Policy Implications”.

7. The reference is somewhat outdated, with fewer references in the last three years.
