# Supplementary material for: Does innovation policy drive rural revitalization in China? - An empirical study based on Chinese rural areas
Source: PLoS One. 2025 Apr 7;20(4):e0311462. doi: 10.1371/journal.pone.0311462 (PMC11975105; doi:10.1371/journal.pone.0311462)
Supplement: Attachment — Submitted filename: To reviewer #2.pdf [file pone.0311462.s004.pdf]

Dear Editors and Reviewers:

Thank you for your letter and for the reviewers' comments concerning our manuscript entitled "How Does Innovation Policy Drive Rural Revitalization in China? - An Empirical Study Based on Rural China".Those comments are all valuable and very helpful for revising and improving our paper, as well as the important guiding significance to our researches. We have studied comments carefully and have made correction which we hope meet with approval. In the revised version, we have highlighted all the changes in yellow. Below are our responses to your comments, listed point by point:

1. The first paragraph of the introduction should briefly present the research questions and the main tenor of the article.

Response: During the revision process, we comprehensively optimized and refined the introduction to clearly present the research problem and the main theme of the article. Specifically, we divided the introduction into two parts: *"Research Background"* and *"Mechanism and Problem Statement"*, aiming to better illustrate the logical starting point and theoretical framework of the study.

In the first part, *"Research Background"*, we focused on highlighting the contemporary context and significance of the research. Specifically, the first and second paragraphs address the pressing challenges faced by rural China, including lagging economic development, population outflow, and imbalanced resource allocation. Additionally, we conducted an in-depth analysis of why China needs to advance the rural revitalization strategy, elaborating on its necessity and urgency from the perspectives of the country's long-term development goals, the importance of narrowing urban-rural disparities, and ensuring social equity. Furthermore, we reviewed the policies and initiatives implemented by the Chinese government to promote rural revitalization, such as targeted poverty alleviation, agricultural modernization, and rural infrastructure development, providing a solid policy context for the subsequent research.

Through these adjustments, we explicitly introduced the research problem and the main theme at the beginning of the introduction, enabling readers to quickly grasp the core focus of the study. At the same time, this part lays a strong foundation for the *"Mechanism and Problem Statement"* section, making the research logic more rigorous and coherent. We believe that these revisions make the introduction more effectively reflect the practical significance and theoretical value of the study, guiding readers seamlessly into the main content of the paper.

2.The literature review is insufficient, especially the literature between innovation policy and rural development was not seen in the article. It is unlikely that this study is the first to study the impact of innovative policies on rural development, and the academic contribution of the article deserves further consideration, which should be based on the literature review.

Response: During the revision process, we made significant adjustments and enhancements to both the introduction and literature review sections to address the reviewer's concern regarding the insufficient coverage of literature on innovation policies and rural development. Our aim was to thoroughly present existing research findings and clearly demonstrate the academic contributions of this study.

Specifically, in the *"Research Background"* section of the introduction, we added a new paragraph focusing on the pivotal role of innovation in advancing rural development in China. This section not only summarizes how innovation technologies and industrial upgrading contribute to optimizing rural economic structures but also analyzes the role of innovation in improving resource allocation,

fostering rural endogenous development, and expanding market opportunities within the context of relevant policy frameworks. This addition provides a clear foundation for the study's relevance. Furthermore, to enhance the depth and breadth of the literature review, we conducted an extensive search of recent key studies on innovation policies and rural development and incorporated the findings into the second section, *"Mechanisms and Problem Statement."*

In the *"Mechanisms and Problem Statement"* section, we introduced two key additions. First, in the first and second paragraphs, we systematically reviewed existing literature on the primary pathways through which innovation impacts rural development, including improving agricultural productivity through innovative technologies, optimizing resource allocation to promote rural industrial diversification, expanding markets and fostering business model innovation to drive economic growth, and enhancing sustainable development and resilience to risks. Second, we cited the latest domestic and international research to emphasize the spillover effects of innovation policies on rural and urban areas, particularly in enhancing rural education, improving healthcare, and upgrading infrastructure. These additions not only enrich the literature review but also provide theoretical support for the study's mechanism design.

Additionally, we explicitly highlight in the introduction and literature review that this study's novelty lies in its focus on China's Innovation City Pilot Policy and its indirect impact on rural development. This specific policy context has not been adequately explored in existing literature. Therefore, this paper conducts an in-depth analysis of the policy characteristics, mechanisms of action, and regional heterogeneity, reflecting the academic significance and practical implications of this research.

3. Lack of theoretical analysis framework and research hypothesis. What is the mechanism and logic of pilot city innovation policy influencing the rural revitalization? How innovation policy act on rural revitalization, and what are the specific transmission paths in this influence? theoretical analysis framework should be constructed before empirical analysis。

Response: During the revision process, we systematically enhanced the theoretical framework of the paper, focusing on how the pilot city innovation policies influence rural revitalization through specific transmission pathways. We also developed a clear theoretical framework to guide the empirical analysis. Specifically, in the *"Mechanisms and Problem Statement"* section of the introduction, we added a detailed theoretical discussion, organized into three key aspects.

First, in the third paragraph, we provided a comprehensive overview of the main components of China's pilot city innovation policies, including strengthening technological research and development, offering tax incentives, fostering talent agglomeration, and optimizing government services. While these policies primarily aim to enhance urban innovation capacity, we emphasized that their effects can spill over to rural areas, serving as a vital driver of rural revitalization.

Second, in the fourth paragraph, we analyzed the specific mechanisms and logical pathways through which innovation policies affect rural revitalization within the context of urban-rural integration. We identified three primary transmission pathways: **technology diffusion** (innovative technologies flow from urban to rural areas, enhancing agricultural productivity and resource efficiency), **factor mobility** (innovation policies attract talent and capital, facilitating efficient allocation and mobility of resources between urban and rural areas), and **economic linkages** (urban innovation outcomes spill over to rural areas through extended industrial chains and expanded markets, promoting rural economic diversification and sustainable development).

Third, in the fifth paragraph, to provide a more intuitive presentation of the theoretical framework, we added a schematic diagram illustrating how innovation policies influence rural revitalization through the three transmission pathways. This diagram clearly outlines the logical sequence from policy implementation to transmission and ultimately to rural socioeconomic development, highlighting key stages and influencing factors. This addition lays a solid foundation for proposing research hypotheses and conducting empirical analysis.

Through these revisions, we not only established a robust theoretical framework but also introduced hypotheses based on the mechanism analysis to guide subsequent empirical research. This framework helps readers better understand the transmission mechanisms and logical pathways of innovation policies' impact on rural revitalization while further emphasizing the academic and practical significance of this study.

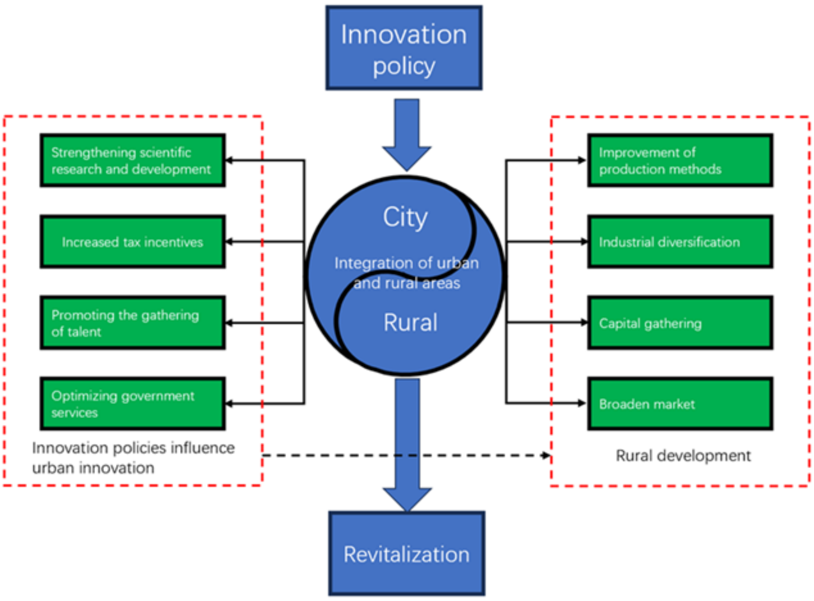

4. The calculation method of the rural revitalization index is not clear. How to establish an evaluation index system (this is an academic contribution stated in the introduction), and how to calculate the index.

Response:

1.Calculation Standards for the Rural Revitalization Index

To clarify the calculation method of the Rural Revitalization Index, this paper adds a detailed explanation of its computation standards in Section 2.2, Rural Revitalization Index and Variable Design, under the second part, Data Description and Model Design. Specifically, the index is constructed as a comprehensive evaluation system based on multiple dimensions, including economic development levels, infrastructure quality, public service delivery, ecological environment status, and social governance capacity, as outlined in Table 1. Additionally, the paper elaborates on the weight allocation and calculation methods for each indicator, ensuring the index's scientific validity and practical applicability.

2.Supplementary Information on the Innovation Pilot Policy

In the same subsection, in paragraph (2), the paper also incorporates the granting dates and corresponding cities of the Innovation Pilot Policy, presented in detail in Table 2. The text provides

a clear explanation of data sources and selection criteria to ensure consistency between policy timeline data and the research design.

5. The explanatory variable is the innovation policy of pilot cities, and in the topic, it is innovation, and the two cannot be equated.One is policy (National Innovation-Driven City Pilot Policy), and the other is innovation ability or activity, which are two research categories.It is suggested that the research object should be specific to innovation policies.
Response:During the revision process, the reviewers' suggestions were adopted by narrowing the scope of the study to focus specifically on the impact of innovation policies on rural revitalization. Accordingly, the title has been revised.

6. The empirical analysis section lacks descriptive statistics of the main variables
Response: During the revision process, descriptive statistics for key variables were added in the Empirical Analysis section, specifically under Baseline Regression, as shown in Table 3.

7. The conclusion part should present the limitations and future research plan.
Response: This paper has been revised in the policy analysis and discussion section, including how to leverage the "urban-rural integration" effect, emphasizing the enhancing roles of innovation in the earlier mediation effect analysis on "communication," "talent," "culture and education," and "policy support," along with the specific methods. Additionally, based on the heterogeneity analysis of different cities, targeted policy recommendations have been proposed. Finally, the paper also highlights areas that have not been studied and suggests directions for future research.

Thank you once again for your valuable feedback. We hope that this article will be accepted. but if you have any further comments or suggestions, please do not hesitate to share them.

Sincerely,
Ying zhige
