# Supplementary material for: Does innovation policy drive rural revitalization in China? - An empirical study based on Chinese rural areas
Source: PLoS One. 2025 Apr 7;20(4):e0311462. doi: 10.1371/journal.pone.0311462 (PMC11975105; doi:10.1371/journal.pone.0311462)
Supplement: Attachment — Submitted filename: reviewers comment.docx [file pone.0311462.s003.docx]

The article has clear research question and well organized structure. Innovative research conclusions were drawn from a normative empirical analysis based on sufficient data amount and reasonable methodology. There still are some problems that need further modification or interpretation.

1. The first paragraph of the introduction should briefly present the research questions and the main tenor of the article.

2.The literature review is insufficient, especially the literature between innovation policy and rural development was not seen in the article. It is unlikely that this study is the first to study the impact of innovative policies on rural development, and the academic contribution of the article deserves further consideration, which should be based on the literature review.

3. Lack of theoretical analysis framework and research hypothesis. What is the mechanism and logic of pilot city innovation policy influencing the rural revitalization? How innovation policy act on rural revitalization, and what are the specific transmission paths in this influence? theoretical analysis framework should be constructed before empirical analysis。

4. The calculation method of the rural revitalization index is not clear. How to establish an evaluation index system (this is an academic contribution stated in the introduction), and how to calculate the index.

5. The explanatory variable is the innovation policy of pilot cities, and in the topic, it is innovation, and the two cannot be equated.One is policy (National Innovation-Driven City Pilot Policy), and the other is innovation ability or activity, which are two research categories.It is suggested that the research object should be specific to innovation policies.

6. The empirical analysis section lacks descriptive statistics of the main variables

7. The conclusion part should present the limitations and future research plan.
