# Supplementary material for: Does innovation policy drive rural revitalization in China? - An empirical study based on Chinese rural areas
Source: PLoS One. 2025 Apr 7;20(4):e0311462. doi: 10.1371/journal.pone.0311462 (PMC11975105; doi:10.1371/journal.pone.0311462)
Supplement: Attachment — Submitted filename: recomendation.docx [file pone.0311462.s005.docx]

The structure, English writing and standardization of the article have been greatly improved in the revision, but the following weak links should be enhanced, mainly in the introduction, theoretical mechanism and standardization of writing.

1.The serial number is not clear, missing 2.1 and 2.1.1

2.Some forms are not standardized，such as “Table 1: Rural Revitalization Evaluation System

needs modification

3.Baseline regression should be Benchmark Regression. Introduce should be introduction

4.In the Introduction part, there is a sentence “Innovation in agriculture, rural areas, and among farmers is not only the foundation of China's stability and well-being but is also critical for other developing countries”. The following relevant literature are all in the Chinese context, and lack of research from other developing countries. It is suggested to expand the scope of the research literature to supplement some studies on the relationship between innovation and agricultural and rural development in other developing countries. It is also suggested to add the relevant literature on the impact of population, policy and science and technology on innovation-enabled rural revitalization.

5.Mechanism analysis should be separated from the introduction. There should be a separate chapter as the theoretical mechanism to construct the theoretical framework and put forward research hypotheses. Additionally, The article lacks theoretical foundation to support it. The theoretical framework Fig.1 cannot clearly reflect the complex mechanism of innovation policy driving Rural Revitalization.

6. There is a repeat of academic contributions in the introduction and conclusion/
