# Supplementary material for: Does innovation policy drive rural revitalization in China? - An empirical study based on Chinese rural areas
Source: PLoS One. 2025 Apr 7;20(4):e0311462. doi: 10.1371/journal.pone.0311462 (PMC11975105; doi:10.1371/journal.pone.0311462)
Supplement: Attachment — Submitted filename: To reviewer(second).docx [file pone.0311462.s006.docx]

Dear Editor and Reviewer,

We feel great thanks for your professional review work on our article. As you are concerned, there are several problems that need to be addressed. According to your nice suggestions, we have made extensive corrections to our previous draft, the detailed corrections are listed below.

1.The serial number is not clear, missing 2.1 and 2.1.1

2.Some forms are not standardized，such as “Table 1: Rural Revitalization Evaluation System

needs modification

Answer(1)-(2): We were really sorry for our careless mistakes. Thank you for your reminder. During this revision process, we have adjusted all formatting according to the LaTeX template provided by the journal. The details can be found in the unmarked version.

3.Baseline regression should be Benchmark Regression. Introduce should be introduction

Answer: In this revision process, in addition to addressing the reviewers' suggested changes, Answer: we also polished the overall language of the article and revised the corresponding expressions. The modifications can be found highlighted in yellow in the marked version.

4.In the Introduction part, there is a sentence “Innovation in agriculture, rural areas, and among farmers is not only the foundation of China's stability and well-being but is also critical for other developing countries”. The following relevant literature are all in the Chinese context, and lack of research from other developing countries. It is suggested to expand the scope of the research literature to supplement some studies on the relationship between innovation and agricultural and rural development in other developing countries. It is also suggested to add the relevant literature on the impact of population, policy and science and technology on innovation-enabled rural revitalization.

Answer: In this revision, we sincerely appreciate the valuable comments. We have checked the literature carefully and added more references on developing country of worldwide into the INTRODUCTION part in the revised manuscript. For example, we incorporated studies on developing countries worldwide, including those in Africa and the Americas. We also expanded the literature review to include research on the roles of population, policies, and technology in innovation-driven rural revitalization, thereby enriching the theoretical framework. These additions can be found in the third and fourth paragraphs of the introduction section.

5.Mechanism analysis should be separated from the introduction. There should be a separate chapter as the theoretical mechanism to construct the theoretical framework and put forward research hypotheses. Additionally, The article lacks theoretical foundation to support it. The theoretical framework Fig.1 cannot clearly reflect the complex mechanism of innovation policy driving Rural Revitalization.

Answer: During this revision, the mechanism analysis has been presented as a standalone chapter, introducing a new theoretical model on "Innovation Policy Driving Rural Revitalization." The pilot policies for innovative cities primarily influence urban areas through smart cities, innovation capacity, and industrial structure, while also promoting urban-rural integration. Based on urban-rural integration, innovation policies further drive rural revitalization through multiple dimensions, including policies, organizations, finance, and talent. The theoretical mechanism diagram (Figure 1) has been redrawn, and additional theoretical elaboration has been included.

6. There is a repeat of academic contributions in the introduction and conclusion/

Answer: The introduction and conclusion have been revised, with all academic contributions articulated as comprehensively as possible in the conclusion section, thereby reducing redundancy.

Thank you once again for your valuable feedback. We hope that this article will be accepted. but if you have any further comments or suggestions, please do not hesitate to share them.

Sincerely,

Ying zhige
